# Enhanced antitumor efficacy of ultrasonic cavitation with up-sized microbubbles in pancreatic cancer

**DOI:** 10.18632/oncotarget.4048

**Published:** 2015-05-08

**Authors:** Pintong Huang, Ying Zhang, Jian Chen, Weihui Shentu, Yu Sun, Zhijian Yang, Tingbo Liang, Shuyuan Chen, Zhaoxia Pu

**Affiliations:** ^1^ Department of Ultrasonography, The Second Affiliated Hospital of Zhejiang University College of Medicine, Hangzhou, Zhejiang Province, P. R. China; ^2^ Department of Surgery, The Second Affiliated Hospital Zhejiang University College of Medicine, Hangzhou, Zhejiang Province, P. R. China; ^3^ Origin Biosciences Inc., Nanjing, Jiangsu Province, P. R. China; ^4^ Baylor Research Institute, Baylor University Medical Center at Dallas, Texas, USA

**Keywords:** ultrasonic cavitation, microbubbles, tumor vasculature, pancreatic cancer, therapy

## Abstract

Ultrasonic cavitation is a novel potential approach for cancer treatment. We optimized the techniques of ultrasonic cavitation to enhance antitumor efficacy in a mouse model with human pancreatic cancer. A polydisperse MB contrast agent formulation (TS-P) with a mean number diameter of 1.9 μm was depleted in small diameter particles by differential centrifugation, producing an “up-sized” size distribution (TS-PL) possessing a mean diameter of 2.9 μm. Mice bearing the XPA-1-RFP pancreatic tumor were treated daily for 3 consecutive days with either up-sized or standard MB. Both treatment cohorts exhibited a significant reduction in tumor volume relative to the untreated control cohort (*P* < 0.05), and TS-PL group has significantly reduction in tumor volume (1215.1± 324.7 mm^3^) compared with standard TS-P group (2131.2±753.4 mm^3^) (*P* < 0.05). The treatment with TS-PL resulted in more tumor cell necrosis and apoptosis than with TS-P. Decreased expression of CD31 and MVD was observed histologically in tumors treated with TS-PL relative to TS-P. This study demonstrates that tuning the size distribution of existing contrast agent products, specifically to reduce the concentration of small MB, is required for enhanced anti-tumor cavitation activity.

## INTRODUCTION

Focused ultrasound has shown great potential as a minimally invasive therapeutic technique. Ultrasound-based histotripsy and thermal ablation are used in current clinical practice, and applications such as thrombolysis, drug and gene delivery, and tumor vasculature destruction are in development [1-7]. Many of these developing therapies have been found to depend upon or be significantly enhanced by the ultrasonic cavitation with microbubbles (MB). At high acoustic pressures (typically beyond those used for imaging), ultrasound causes acoustic cavitation and microbubble destruction, and can produce extremely high mechanical stresses [8-10] over a very localized region. MB-enhanced ultrasound has a potiential both in improving the targeting and the efficacy of drug delivery for cancer. MB-mediated ultrasonic cavitation has been reported for treating central nervous system leukemia with ultrasonic MB loading drug, eliciting tumor perfusion reduction, apoptosis, significant growth inhibition, and necrosis in gliomas [11], reducing DU-145 prostate xenograft with delivery of p53 and RB gene [12] and treating human pancreatic cancer using combined ultrasound, microbubbles, and gemcitabine [13]. We have previously shown that the application of ultrasonic cavitation with commercially available MB imaging agents demonstrated the anti-vascular and anti-tumor effects for colon cancer [14, 15].

Cavitation is the response of gas bubbles in liquid after oscillating to an acoustic field [8, 16, 17]. Until now, most studies have used lipid-based MB that are formed by self-assembled monolayer phospholipids and are responsive to ultrasound [18]. The MBs are highly polydisperse agents with bubble diameters ranging from submicrons to 10 mm [19]. The high polydispersity is a consequence of the emulsification methods used to generate MB in high quantity, such as sonication, shaking and milling. Feshitan et al. [20] found that lipid-coated MB formed by sonication was multi-modal in size, with most bubbles between 1-2 μ m and distinct subpopulations at 4-5 μ m and 6-8 μm in diameter. The amplitude of the oscillations during ultrasound imaging or cavitation experiences a resonance that depends on the size of the bubble and frequency of the sound [21-23]. Choi et al. [24] reported that the focused ultrasound induced blood-brain barrier opening was dependent on both the size distribution in the injected MB volume and the brain region targeted. However, the relative contribution of each size class of MB to the acoustic response, the oscillation amplitude and vasculature destruction in tumor are unclear.

Pancreatic cancer is a highly treatment-resistant disease. Improved therapeutics for pancreatic cancer is still urgently needed. Application of MB-mediated ultrasonic cavitation in pancreatic cancer is very limited. In this study we examined anti-tumor effect of MB-mediated ultrasonic cavitation in a xenograft mouse model of pancreatic cancer. We aimed to compare the cavitation-mediated anti-tumor effect of a MB imaging agent with or without depletion of small MB. Commercial MB formulations are polydisperse, with MB of diameter asymmetrically distributed between 1∼8 μm. Although polydispersity may be a benefit in the context of contrast ultrasound imaging, this may not be the case for cavitation therapy. In the current study, MB were administered to mice at the same number dose (number of MB per dose), and treated with identical acoustic settings. This design enabled us to isolate the role of MB diameter, independent of changes in shell or gas composition typically found between different contrast agent preparations.

## RESULTS

### Microbubble (MB) size distribution

**Figure 1:**
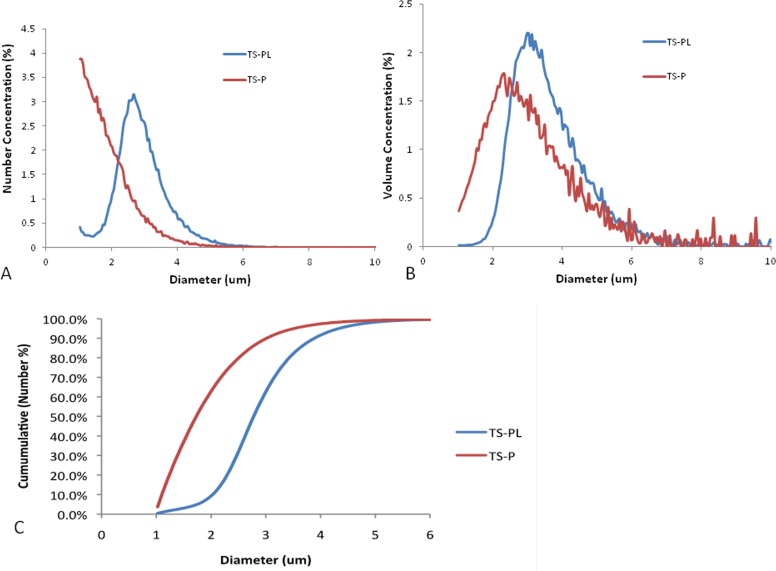
Size distributions of standard TS-P and up-sized TS-PL **A.** Number concentration (%) versus MB diameter. **B.** Volume concentration (%) versus MB diameter, and **C.** cumulative number concentration.

The size distributions of standard TS-P and up-sized TS-PL in both volume and number mode are illustrated in Figure 1. The up-sized TS-PL had fewer MB of diameter < 2 μm (Figure 1A). The mean diameter by number of standard TS-P was 1.9 μm, that of up-sized TS-PL was 2.9 μm (Figure 1B). The cumulative size distribution presented in Figure 1C illustrates the difference in concentration of small (1-2 μm diameter) MB between the formulations. This data is further described by Table 1, in which the min and max values between 4 separate measurements are presented.

**Table 1:** Summary of microbubble size ranges for TS-P and TS-PL preparations

Diameter(um)	TS- P(%)	TS-PL(%)
1.0	3.6-3.8	0.3-0.6
1.5	36.9-37.9	2.6-3.4
2.0	62.1-63.8	8.9-9.8
2.5	79.3-81.3	32.7-33.4
3.0	89.4-91.1	64.2-64.2
3.5	94.2-95.6	81.7-82.3
4.0	96.9-97.9	91.3-92.1

Note: Data are reported as percent of total microbubbles having diameter less than the specified value, over 1.0 to 4.0 μm. Data are expressed as min and max values from n=4 measurements.

### Effect of cavitation with MB on tumor vascular perfusion

**Figure 2:**
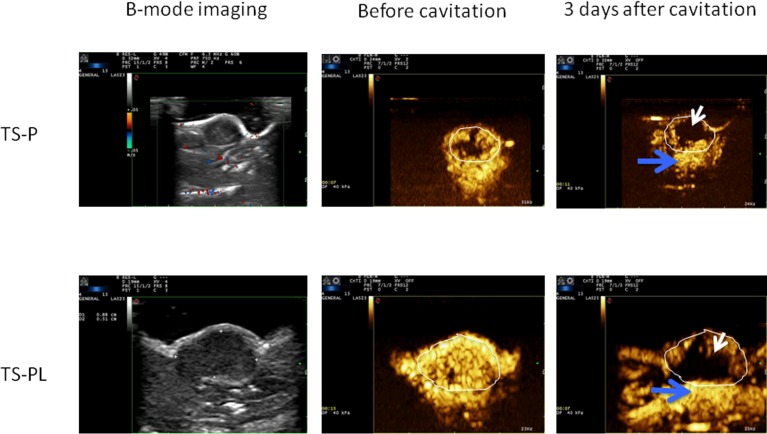
Effect of Up-sized MB on tumor vascular perfusion Tumor-bearing animals were treated with ultrasonic cavitation when xenograft tumors had reached 120 mm^3^ as described in the MATERIALS AND METHODS. Representative B-mode and contrast-enhanced ultrasound imaging of the tumors before and after treatment. Outlined circle indicates tumor. White arrows depict regions of absent blood flow.

The tumor vascular perfusion was assessed by contrast-enhanced ultrasound imaging (CEUS) immediately before and after three ultrasonic cavitation treatments and quantitated with the standard vascular perfusion parameters RBV and RBF. In Figure 2 the presence of contrast (and hence vascular perfusion) is color coded in shades of orange. Ultrasonic cavitation treatment with either MB preparation significantly reduced blood flow within the tumor relative to before treatment. Perfusion in the underlying non-tumor tissue (Blue arrows in Figure 2) remains after treatment, suggesting that the anti-vascular effect is tumor-specific.

Quantified vascular perfusion parameters are given in Table 2. A statistically significant decrease for vascular perfusion parameters (RBV and RBF) was found after three treatments for standard TS-P and up-sized TS-PL treated tumors. There was no significant difference in any of these derived CEUS parameters between TS-P and up-sized TS-PL treated tumors.

**Table 2:** Quantitation of microbubble infusion into the tumor before and after cavitation

		Pre-treatment	3 days after cavitation	*P* value*
TS-P	RBV(mL)	452.47±46.15	321.67±55.34	*P* = 0.005
	RBF(mL/s)	46.64±6.47	31.19±5.44	*P* = 0.001
TS-PL	RBV(mL)	478.61±55.23	224.27±58.76	*P* = 0.001
	RBF(mL/s)	47.27±9.23	23.43±5.49	*P* = 0.003

RBV: regional blood volume; RBF: regional blood flow.

* *P* value, when compared with that before cavitation.

### Effect of up-sized MB on xenograft tumor growth

**Figure 3:**
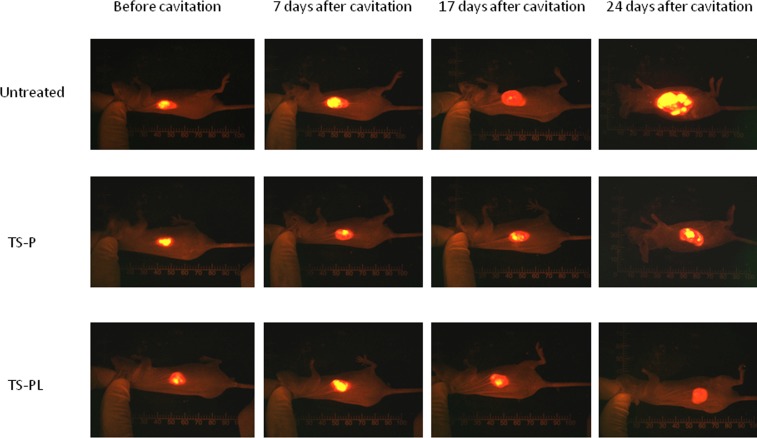
Inhibitory effect of Up-sized MB on xenograft tumor growth Whole body fluorescence imaging was performed to measure tumor size at different time points during the study as described in the MATERIALS AND METHODS. Sequential in vivo real-time fluorescence imaging of tumor progression after ultrasonic cavitation.

**Figure 4:**
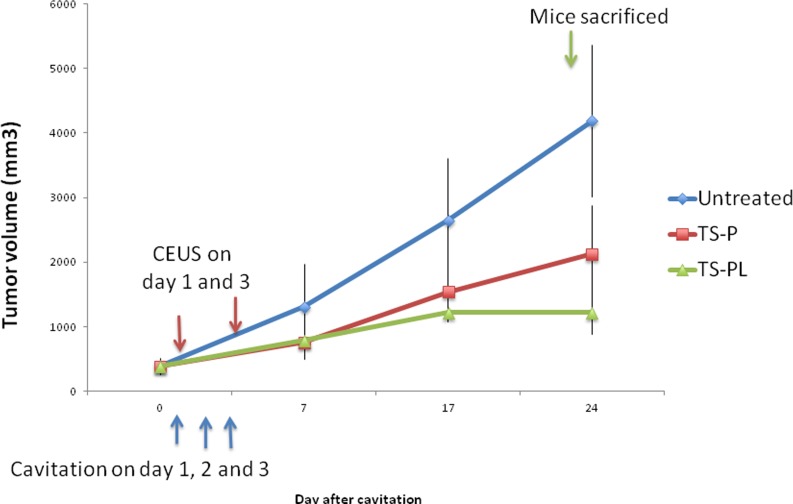
Inhibitory effect of Up-sized MB on xenograft tumor growth Whole body fluorescence imaging was performed to measure tumor size at different time points during the study as described in the MATERIALS AND METHODS. Tumor growth curves after treatments.

The tumor growth inhibition was evaluated by non-invasive fluorescence imaging. Tumor size was measured on day 0, 7, 17 and 24 following ultrasonic cavitation with standard and up-sized MB. Representative fluorescence images Figure 3, and tumor growth curves in Figure 4. Significant inhibition in tumor growth relative to untreated was observed for both MB preparations at day 7, and persisted throughout the study. By day 24, tumors treated with TS-PL (1215.1± 324.7 mm^3^) were significantly (*P* < 0.05) smaller than those treated with standard TS-P (2131.2±753.4 mm^3^).

### Effect of up-sized MB on tumor histology and vasculature

**Figure 5:**
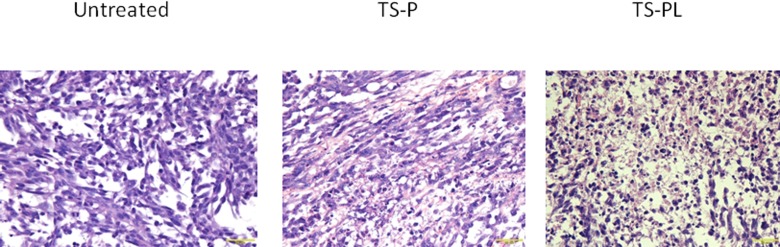
Effect of Up-sized MB on tumor histology H&E staining of the tumor tissue were performed as described in the MATERIALS AND METHODS. Representative images of H&E staining for the samples from untreated, standard TS-P and up-sized TS-PL MB (400× magnification, scale bar 20 μm).

As shown in Figure 5, XPA-1 pancreatic tumor tissue in the untreated group reveals nests of flaky, cluster-like and diffusely infiltratrative cells. Tumor cells were irregularly arranged with an irregular morphology. The nuclei were large and deeply stained, with obvious atypia and rare nucleolus, as would be expected for this cancer model. The tumor tissue of the TS-P group showed varying degrees of degenerative changes and their cytoplasm was swollen, with cyto­plasmic vacuoles of various sizes. The tumor tissue of TS-PL group showed scattered tumor cell necrosis. The residual tumor cells showed obvious degenerative deformation, chromatin condensation, with cell debris seen around necrotic tumor cells.

**Figure 6:**
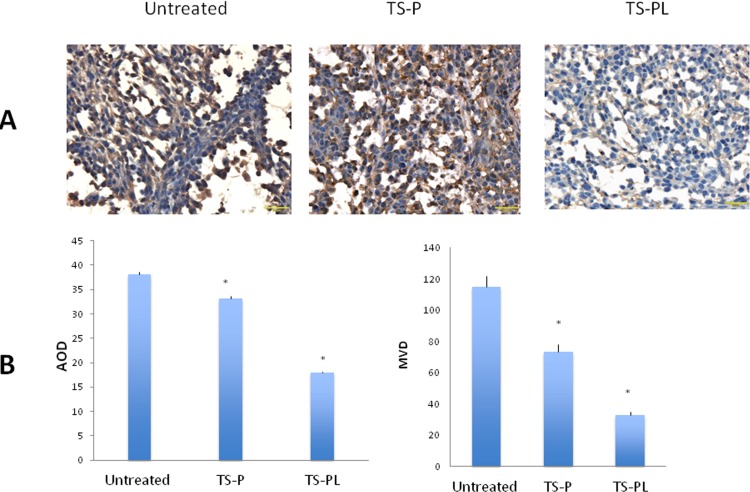
Effect of Up-sized MB on tumor angiogenesis Immunohistochemical anti-CD31 staining of the tumor tissue was performed as described in the MATERIALS AND METHODS. **A.** Representative CD31 immunohistochemical images (400× magnification, scale bar 20 μm). Immunoreactivity for CD31 was present when the cytoplasm of cells stained brown. **B.** Quantitation of the expression of CD31 by average optical density (AOD) and microvessel density (MVD). *P < 0.01, when compared with untreated group.

Immunohistochemical staining with CD31was performed to assess the effect of up-sized MB on tumor vasculature. More decreased CD31 expression and MVD were found in up-size TS-PL than standard TS-P treated group as compared to untreated control, suggesting stronger anti-vascular effect for up-size MB (Figure 6A & 6B).

### Effect of up-sized MB on tumor apoptosis

**Figure 7:**
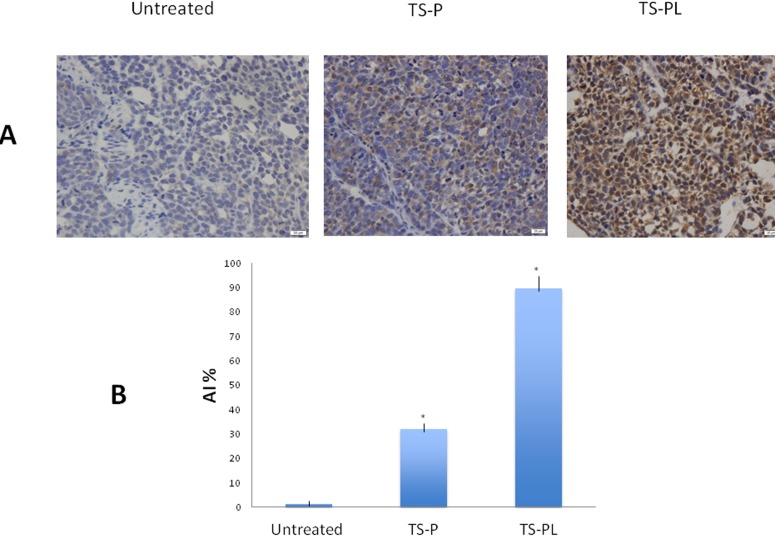
Effect of Up-sized MB on tumor apoptosis The tumor sections were stained with TUNEL to measure apoptosis of tumor cells as described in the MATERIALS AND METHODS. **A.** Representative TUNEL images for cell apoptosis (400× magnification, scale bar 20 μm). **B.** Quantitation of cell apoptosis by apoptotic index (AI). *P < 0.01, when compared with untreated group.

The effect of up-sized MB on tumor apoptosis was analyzed by TUNEL staining. TUNEL-positive cells were counted only in regions of intact tumor, being careful to avoid the central necrosis typically observed in xenografts. The number of apoptotic cells in 3 random fields from 3 different tumors in each group was counted, and the apoptotic index and representative fields of view from each group are shown in Figure 7. Significant high apoptotic index was found in mice treated with TS-P or TS-PL as compared to untreated group. TS-PL showed higher apoptotic index than TS-P.

## DISCUSSION

The unique physiological barriers presented by the tumor environment and associated vasculature impose a limit to the therapeutic efficacy of systemically delivered anticancer agents [26]. Several strategies for specifically targeting the tumor have previously been applied in an effort to develop efficient and noninvasive ways of delivering anticancer agents and optimizing therapeutic protocols [27]. Therapeutic ultrasound using MB-mediated ultrasonic cavitation has demonstrated great potential as a means for achieving targeted disruption of tumor vasculature [4-6]. However, the interaction between MB-mediated ultrasonic cavitation and surrounding blood vessels is a complicated phenomenon, which still requires extensive investigations from all perspectives [28, 29]. In the present study, we focused primarily on the effect of initial MB size on anti-tumor efficacy.

As illustrated in Figure 1, although both sizes of MB exhibited polydispersity, the volumetric size distribution varied across different diameter ranges. The up-sized TS-PL was enriched in MB of diameter > 2 μm, whereas standard TS-P had a relatively fewer MB > 2 μm (Table 1). Fluorescence imaging of the RFP-expressing tumors, which enabled sensitive visualization of tumor dimensions, revealed significantly greater tumor growth inhibition by up-sized MB relative to smaller standard MB at day 24. Significant decrease in microvessel density and induction of apoptosis for treated animals were observed at this same timepoint and were more pronounced for the TS-PL treatment group than for TS-P. Choi [25] showed large MB are better for BBB opening. Our study is first report to demonstrate better anti-tumor effect of large MB in tumor mouse model.

Contrast-enhanced ultrasound performed at day 3 revealed a significant change in intra-tumor blood flow and volume between untreated and treated tumors. However, we did not detect a difference between MB preparations at this timepoint. It may be the case that changes in vascular perfusion need some time to occur; alterations in MVD found at day 24 supports this hypothesis. Alternatively, our finding may reflect a limitation in the sensitivity of CEUS, which may not be able to distinguish subtle changes in perfusion between the treatment conditions. The non-invasive nature of CEUS lends itself to repeated use, and performing CEUS at multiple timepoints throughout the study may reveal further insights into how vascular perfusion changes during the course of therapy.

The pancreatic tumors treated with up-sized MB-mediated ultrasonic cavitation showed some char­acteristic pathological changes that were different from those of standard MB. Ashush *et al.* [30] observed the following morphological changes after US cavitation: cell shrinkage, vacuole formation, chromatin condensation, karyorrhexis and the formation of apoptotic bodies. Our study found that up-sized MB-mediated ultrasonic cavitation caused higher degrees of degenerative deformation, chromatin condensation of tumor cells and more tumor cell necrosis as compared to standard size MB treated tumor.

Tumor angiogenesis is of vital importance to the growth and metastasis of solid tumors and is featured with a defective, leaky and fragile vascular construction. MB enhanced ultrasound cavitation is capable of mechanical disruption of small blood vessels depending on effective acoustic pressure amplitude [31]. This study found that more decreased CD31 expression was found in up-size MB than standard MB treated group, suggesting stronger anti-angiogenesis effect for up-size MB.

To further clarify more effective anti-cancer mechanism of up-size MB mediated ultrasound cavitation, we stained tumor sections with TUNEL to measure apoptosis of tumor cells. We found that ultrasound cavitation with up-size MB caused significant more apoptosis of tumor cells than standard MB, indicating the degree of tumor cell apoptosis is associated with microbubble size in ultrasonic cavitation treatment.

Our study does not clearly identify the mechanism behind our observed enhanced efficacy of larger MB in cavitation therapy. We hypothesize that the larger MB may have a greater probability of interacting with capillaries while oscillating. Under same ultrasound operating frequency, pressure of the ultrasound wave and bubble shell thickness, The MB with the larger initial radius may absorb more energy from the acoustic field and has the greater bubble expansion and compression ratio that result in higher oscillation amplitudes to cause more damage to blood vessels [32]. Therefore, more than 2 μm components presented in up-sized TS-PL MB might have contributed to the significantly different anti-vascular effect and tumor growth inhibition.

There are several limitations to the present study. There was only one up-sized MB used in this study. A wider panel of MB diameters and ultrasonic cavitation conditions should be investigated to ascertain the optimal ultrasonic cavitation efficacy. Another limitation is that the potential for damage to normal tissues was not analyzed for the up-sized MB. The potential for unwanted ultrasound-related bioeffects has been reported in various models [33-35], and represents a potential hurdle for application of this technology in a clinical setting. A rigorous study to evaluate off-target effects pertaining to the therapy is warranted.

Current treatment options for pancreatic cancer are limited, and despite decades of effort prognosis remains extremely poor. A clinical study for safety of combining ultrasound MB and chemotherapy to treat liver metastases from gastrointestinal tumors and pancreatic carcinoma has been published (https://clinicaltrials.gov/). Despite the procedural complexity and potential regulatory hurdles, the cavitation-based treatment proposed here warrants further development.

This study indicated that the up-sized MB might be considered more efficient for tumor inhibition compared with smaller standard MB and by combining low frequency US with up-sized MB, cavitation effects may be intensified to achieve more effective anti-tumor potential.

## MATERIALS AND METHODS

### Microbubbles

Targestar^®^-P (Targeson; San Diego, CA) was used in all studies. Targestar-P (TS-P) is a lipid encapsulated decafluorobutane (C_4_F_10_) microbubble (MB), and is commercially available for use as an imaging agent in life science research. Targestar-P is a polydisperse formulation, containing MB of diameter between 1 and 8 um. An “up-sized” preparation of Targestar-P was prepared by differential centrifugation, as in Shekhar et al (18). Following centrifugation, MB were packaged in depyrogenated glass vials under a headspace of decafluorobutane and stored at 4-8°C. MB size distribution and concentration was assessed by electrozone sensing, using a Coulter Multisizer 3 (Beckman Coulter, Fullerton, CA, USA), which measures MB in the range 0.6–18 μm.

### Cell culture

A human pancreatic cancer cell line XPA-1 transfected with red fluorescence protein gene (XPA-1-RFP) was obtained from AntiCancer, Inc., (San Diego, CA). Cells were cultured in RPMI 1640 (GIBCO Life Technologies, New. York, NY) containing 10% heat-inactivated fetal bovine serum (FBS, Hyclone, Logan, UT) at 37°C in 5% CO_2_ saturated humidity. All media was supplemented with penicillin/streptomycin (Gibco-BRL, Grand Island, NY).

### Xenograft tumor model

18 BALB/C male nude mice aged 4-6 weeks and weighing 20-25 g, were purchased from the Beijing Kelihua laboratory animal center (Beijing, P.R.China). All mice were maintained in a HEPA-filtered environment at 24-25°C and humidity was maintained at 50-60%. All animals were fed with autoclaved laboratory rodent diet. Animal experiments were approved by the Animal Committee of Nanjing Origin Biosciences, China.

XPA-1-RFP tumors were established by subcutaneously injecting 5×10^6^ XPA-1-RFP cells in the flank of nude mice. Pancreatic tumors, grown subcutaneously in nude mice, were harvested at the exponential growth phase and resected under aseptic conditions. Strong RFP expression of the XPA-1-RFP tumor tissue was confirmed by fluorescence microscopy. Necrotic tissues were removed and viable tissues were cut with scissors and minced into 1-mm^3^ pieces. Animals were anesthetized prior to surgery by injection of 0.02 ml of solution of 50% ketamine (100 mg/ml), 38% xylazine (100 mg/ml), and 12% acepromazine maleate (10 mg/ml) Two pieces of tumor fragment were transplanted to the flank of nude mice with 8-0 surgical sutures. All surgical procedures and animal manipulations were conducted aseptically under HEPA-filtered laminar-flow hoods with a ×8 surgical microscope (Shanghai Precision Instruments, YZ20P5, Shanghai, China).

### Administration of ultrasonic MB

Eighteen tumor-bearing mice were randomly divided into 3 groups of 6 mice once the average tumor size had reached 100 mm^3^. Mice were anesthetized with ketamine, acepromazine and xylazine and then placed in a supine position on a heated stage. A dose of 1×10^8^ MB in 70 μl per mouse was administered by retro-orbital injection with a 27gauge needle as in our previous study [16]. Group 1 served as an untreated control and didn't received MB and ultrasonic cavitation treatment. Group 2 received standard MB (TS-P). Group 3 received up-sized MB (TS-PL).

### Ultrasonic cavitation

1 min after MB administration, ultrasound was applied locally to the tumors of anesthetized mice. The animals in Group 2 and 3 received ultrasonic cavitation treatments daily over three days under identical acoustic conditions as in our previous study [16]. The transducer from a sonicator equipped with a 1cm^2^ transducer cone tip (Haiying Medical Electronic Instrument Company, Wuxi, China) was placed over the tumor and coupled using acoustic coupling gel. Ultrasonic cavitation was performed at a frequency of 238 KHz, 400 mV, 0.5 MPa 60-second sonication duration, 10 pulses with 10-ms pulse length and 50% duty cycle.

### Contrast-enhanced ultrasound imaging

Contrast-enhanced ultrasound imaging (CEUS) was used to non-invasively assess tumor vasculature during the study as in our previous study [16]. CEUS was performed before cavitation (Day 1) and day 3 after the final cavitation treatment. CEUS was performed on a Mylab90 ultrasound scanner with 4.0MHz-11.0 MHz LA332 linear transducer (Esaote, Genova ITALY). The transducer was coupled to the skin covering the tumor with acoustic coupling gel. Imaging was performed in CnTI mode at a mechanical index (MI) of 0.04 and transmission frequency of 8 MHz. Imaging gain settings were optimized and held constant during the experiment. Immediately after injection of microbubbles (Targestar-P), ultrasound images were captured over to obtain a signal from the tumor tissue as well as from adherent and freely circulating microbubbles. Digital raw data were stored as cine loops up to 2 minutes for analysis. One board-certified abdominal radiologists (CZ with 15 years of CEUS experience) blinded to the treatment groups reviewed and analyzed the data offline using the perfusions software QONTRAST (Bracco, Italy). A region of interest (ROI) was drawn freehand around the peripheral margin of the tumor using an on-screen cursor, taking care to avoid the surrounding tissue. A time-intensity curve (TIC) for the selected tumor region of interest was plotted by the software, and the following parameters were calculated by the software: Regional blood volume (RBV), which is proportional to the area under the time-intensity curve; Regional blood flow (RBF), which is the ratio of the RBV to MTT (Mean transit time); and area under the time-intensity curve (AUC).

### *In vivo* fluorescence imaging

Upon completion of the ultrasonic cavitation treatments, the tumor bearing mice were monitored by real-time whole-body fluorescence imaging for tumor growth. Imaging was performed before cavitation (day 0) and on day 7, 17 and 24. Tumor dimensions were measured and volume was calculated using the formula (L x W^2^) x ½, where W and L represent the perpendicular minor dimension and major dimension, respectively. A fluorescence stereo microscope (MZ650; Nanjing Optic Instrument Inc. China) equipped with D510 long-pass and HQ600/50 band-pass emission filters (Chroma Technology, Brattleboro, VT) and a cooled color charge-coupled device camera (Qimaging, BC, Canada) was used as in our previous study [16]. Selective excitation of RFP was produced through an illuminator equipped with HQ520/40 and HQ570/40 excitation band-pass filters (Chroma Technology, Brattleboro, VT). Images were processed and analyzed with the use of IMAGE PRO PLUS 6.0 software (Media Cybernetics, Silver Spring, MD).

### Histology and immunohistochemistry

At the end of the study, all mice were sacrificed and the tumors were surgically excised. Tumor tissues were fixed in 10% buffered formalin and parafﬁn-embedded. Hematoxylin and eosin (H&E) staining was performed on paraffin-embedded tumor tissues with a slice thickness of 6 μm for histological study. For immunohistochemistry, sections were incubated with primary antibody against CD31 (BD Biosciences, San Diego, CA) overnight at 4°C after permeabilization with a solution of 0.1% sodium citrate and 0.1%Triton X-100 and blocking with 10% rabbit serum. After washing in PBS, the slices were incubated with horseradish peroxidase-labeled secondary antibody (1:200, Maixin Bio-Tech Co., Ltd, Fuzhou, China) for 30 minutes at room temperature. After color development using diaminobenzidine (Maixin Bio-Tech Co., Ltd), the slices were counterstained in hematoxylin and mounted with a neutral resin medium. The whole slide was first viewed at 100-times magnification in order to identify a “hot spot” representing the area of the highest vessel density. The field was then switched to ×400 magnification for analysis. For each slide, the microvessel density (MVD) was calculated as the average number of CD31+ vessels in 4 fields of view.

### TUNEL analysis for detection of apoptosis

Apoptosis of the tumor cells following cavitation therapy was determined by TUNEL staining, using a commercially available kit (In Situ Cell Death Detection Kit, POD; Roche, Germany). Tumor sections were deparaffinized and dehydrated according to standard protocols. Tissue sections were incubated with Proteinase K working solution for 30 min at 21-37°C. The slides were then washed with PBS (pH 7.2-7.6) twice. The positive control was incubated with DNase I for 10 min at 15-25 °C. The negative control was incubated with label solution (without terminal transferase) instead of the TUNEL reaction mixture. The slides were then washed with PBS (pH 7.2-7.6) three times. Converter-POD was added on slides and incubated in a humidified chamber for 30 min at 37°C. The slides were then washed with PBS (pH 7.2-7.6) three times. The DAB substrate was added on slides and incubated for 10 min at 15-25°C. The slides were then washed with PBS (pH 7.2-7.6) three times. The slides were mounted and analyzed by microscope (Olympus, Melville, NY, USA). The apoptotic index (AI) was calculated by counting the number of TUNEL+ nuclei visible on high-power-field 40Å∼ objective in at least 3 fields/sample and expressing the results as percentage of the total number of cells in the same fields. Apoptotic cells were recognized by the appearance of brown or tan stained nuclei.

### Statistical analysis

Data are expressed as means ± standard deviation. Student's *t*-tests were performed to make comparisons between the two MB preparations used in this study. A *p* < value, 0.05 was considered significant.
